# MetaboAnalyst 6.0: towards a unified platform for metabolomics data processing, analysis and interpretation

**DOI:** 10.1093/nar/gkae253

**Published:** 2024-04-08

**Authors:** Zhiqiang Pang, Yao Lu, Guangyan Zhou, Fiona Hui, Lei Xu, Charles Viau, Aliya F Spigelman, Patrick E MacDonald, David S Wishart, Shuzhao Li, Jianguo Xia

**Affiliations:** Institute of Parasitology, McGill University,Sainte-Anne-de-Bellevue, Quebec, Canada; Department of Microbiology and Immunology, McGill University, Montreal, Quebec, Canada; Institute of Parasitology, McGill University,Sainte-Anne-de-Bellevue, Quebec, Canada; Institute of Parasitology, McGill University,Sainte-Anne-de-Bellevue, Quebec, Canada; Institute of Parasitology, McGill University,Sainte-Anne-de-Bellevue, Quebec, Canada; Institute of Parasitology, McGill University,Sainte-Anne-de-Bellevue, Quebec, Canada; Department of Pharmacology and Alberta Diabetes Institute, University of Alberta, Edmonton, Alberta, Canada; Department of Pharmacology and Alberta Diabetes Institute, University of Alberta, Edmonton, Alberta, Canada; Departments of Biological Sciences and Computing Science, University of Alberta, Edmonton, Alberta, Canada; The Jackson Laboratory for Genomic Medicine, Farmington, CT, USA; University of Connecticut School of Medicine, Farmington, CT, USA; Institute of Parasitology, McGill University,Sainte-Anne-de-Bellevue, Quebec, Canada; Department of Microbiology and Immunology, McGill University, Montreal, Quebec, Canada

## Abstract

We introduce MetaboAnalyst version 6.0 as a unified platform for processing, analyzing, and interpreting data from targeted as well as untargeted metabolomics studies using liquid chromatography - mass spectrometry (LC–MS). The two main objectives in developing version 6.0 are to support tandem MS (MS2) data processing and annotation, as well as to support the analysis of data from exposomics studies and related experiments. Key features of MetaboAnalyst 6.0 include: (i) a significantly enhanced Spectra Processing module with support for MS2 data and the asari algorithm; (ii) a MS2 Peak Annotation module based on comprehensive MS2 reference databases with fragment-level annotation; (iii) a new Statistical Analysis module dedicated for handling complex study design with multiple factors or phenotypic descriptors; (iv) a Causal Analysis module for estimating metabolite - phenotype causal relations based on two-sample Mendelian randomization, and (v) a Dose-Response Analysis module for benchmark dose calculations. In addition, we have also improved MetaboAnalyst's visualization functions, updated its compound database and metabolite sets, and significantly expanded its pathway analysis support to around 130 species. MetaboAnalyst 6.0 is freely available at https://www.metaboanalyst.ca.

## Graphical Abstract

**Graphical Abstract:**
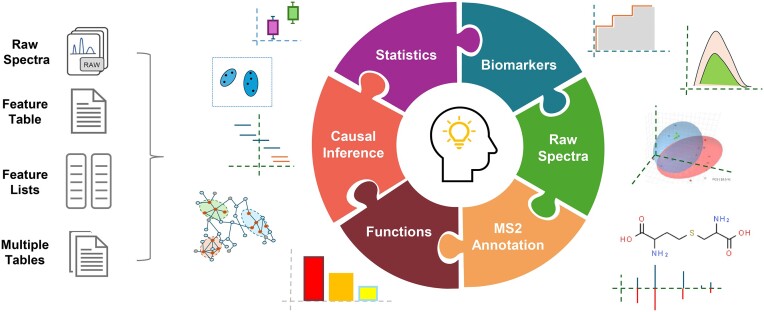

## Introduction

Metabolomics involves the comprehensive study of all small molecules in a biological system. It has diverse applications ranging from basic biochemical research to clinical investigation of diseases, food safety assessment, environmental monitoring, etc. (1–5). User-friendly and easily accessible bioinformatics tools are essential to deal with the complex data produced from metabolomics studies. MetaboAnalyst is a user-friendly, web-based platform developed to provide comprehensive support for metabolomics data analysis (6–10). The early versions (1.0–3.0) focused primarily on supporting statistical and functional analysis of targeted metabolomics data. Increasing support for untargeted metabolomics data from liquid chromatography–mass spectrometry (LC–MS) experiments have been gradually introduced in more recent versions of MetaboAnalyst. For instance, version 4.0 implemented a new module to support functional analysis directly from LC–MS peaks, while version 5.0 added an auto-optimized LC–MS spectral processing module that works seamlessly with the functional analysis module. A detailed protocol on how to use different modules for comprehensive analysis of untargeted metabolomics data was published in 2022 (11). According to Google Analytics, the MetaboAnalyst web server has processed over 2 million jobs, including 33 000 spectral processing jobs over the past 12 months. Many of these jobs are associated with untargeted metabolomics and exposomics studies.

Untargeted metabolomics data generated from high-resolution LC–MS instruments are typically characterized by thousands of peaks with unknown chemical identities. To assist with compound identification, tandem MS (called MS/MS or MS2) spectra are often collected from pooled QC samples during the experiments (12). The two commonly used MS2 methods are data-dependent acquisition (DDA) and data-independent acquisition (DIA), with sequential window acquisition of all theoretical mass spectra (SWATH) being a promising special case of the latter. DDA data usually have clear associations between the precursor ions and the corresponding MS2 spectra, while DIA data generally require deconvolution of the MS2 data to reconstruct associations with their precursor ions (13). Incorporating MS2 processing and annotation into untargeted metabolomics workflows can greatly improve compound annotations and functional interpretation.

Exposomics is an emerging field centered on profiling the complete set of exposures individuals encounter across their lifespan, which often involves MS analysis of chemical mixtures traditionally rooted in toxicology and public health (4). Untargeted LC–MS based metabolomics is increasingly applied to exposomics and toxicology studies. Exposomics data from human cohorts is often associated with complex phenotypic data due to their observational nature. This requires more sophisticated data analysis and visualization methods that can take into consideration of multiple factors or covariates. Exposomics studies typically produce long lists of potential biomarkers that are significantly associated with phenotypes of interest. Identification of causal links from this large number of metabolite-phenotype relations is a natural next step. It has become possible recently with the availability of many metabolomic genome-wide association studies (mGWAS) that link metabolites and genotypes (14–16). By integrating mGWAS data with comparable GWAS data that associate genotypes with various phenotypes (17), we can now estimate causal relationships between a metabolite and a phenotype of interest through Mendelian randomization (MR) (18). Dose-response experiments are often performed to further quantify cause-and-effect relationships. The experiments are often conducted at multiple dose levels using *in vitro* assays or animal models to calculate dose-response curves for risk assessment of chemical exposures (19–21).

To address these emerging needs from both the metabolomics and exposomics communities, we have developed MetaboAnalyst version 6.0. This version includes many key features:

- A significantly enhanced spectra processing workflow with the addition of asari algorithm for LC–MS spectra processing (22), as well as support for MS2 (DDA or SWATH-DIA) data processing.
- A new module for MS2 spectral database searching for compound identification and results visualization.
- A new module for causal analysis between metabolites and phenotypes of interest based on two-sample MR (2SMR).
- A new module for dose-response analysis including dose-response curve fitting and benchmark dose (BMD) calculation.
- A new module for statistical analysis with complex metadata;
- A number of other important updates including: improved functional analysis of untargeted metabolomics data by integrating MS2-based compound identification; updated compound database, pathways and metabolite sets; as well as improved data visualization support across multiple modules.

MetaboAnalyst 6.0 is feely accessible at https://www.metaboanalyst.ca, with comprehensive documentations and updated tutorials. To better engage with our users, a dedicated user forum (https://omicsforum.ca) has been operational since May 2022. To dates, this forum contains >4000 posts on ∼700 topics related to different aspects of using MetaboAnalyst.

## Overall design and workflow of MetaboAnalyst 6.0

**Figure 1.**
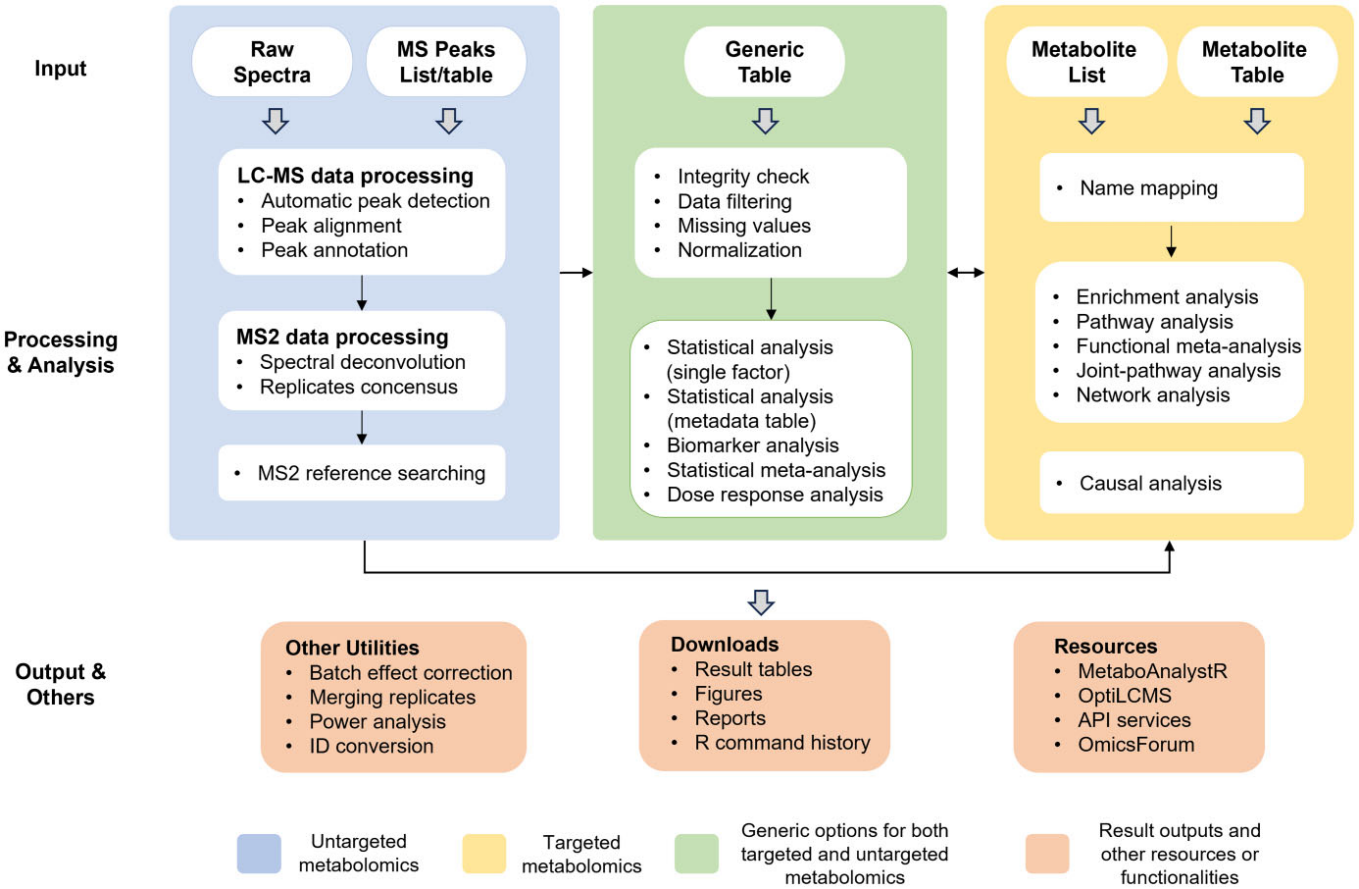
MetaboAnalyst 6.0 workflow for targeted and untargeted metabolomics data. Multiple data input types are accepted. Untargeted metabolomics inputs require extra steps for spectra processing and peak annotation. The result table can be used for statistical and functional analysis within a consistent workflow in the same manner as for targeted metabolomics data.

MetaboAnalyst 6.0 accepts a total of five different data types across various modules encompassing spectra processing, statistical analysis, functional analysis, meta-analysis, and integration with other omics data. Once the data are uploaded, all analysis steps are conducted within a consistent framework including data integrity checks, parameter customization, and results visualization (Figure 1). Some of the key features in MetaboAnalyst 6.0 are described below.

## Supporting asari and MS2 spectra in LC–MS spectra processing workflow

LC–MS spectra processing remains an active research topic in the field of untargeted metabolomics. Many powerful tools have been developed over time, including XCMS (23), MZmine (24), MS-DIAL (13) and asari (22). In addition to using different peak detection algorithms, most tools require manual parameter tuning to ensure good results. Such practice often leads to results that vary significantly (25). To mitigate this issue, MetaboAnalyst 5.0 introduced an auto-optimized LC–MS processing pipeline to minimize the parameter-related effects (10,26). The asari software has introduced a set of quality metrics, concepts of mass tracks and composite mass tracks and new algorithmic design to minimize errors in feature correspondence. It requires minimal parameter tuning while achieving much faster computational performance (22). The asari algorithm is now available in the LC–MS spectra processing options, alongside the traditional approaches.

MS2 spectra processing and metabolite identification are important components of untargeted metabolomics. It is now recognized that MS2 spectral deconvolution is necessary to achieve high-quality compound identification results for both DDA and SWATH-DIA data (27–29). MetaboAnalyst 6.0 offers an efficient, auto-optimized pipeline for MS2 spectral deconvolution. The DDA data deconvolution method is derived from the DecoID algorithm (28), which employs a database-dependent regression model to deconvolve contaminated spectra. The SWATH-DIA data deconvolution algorithm is based on the DecoMetDIA method (29), with the core algorithm re-implemented using a Rcpp/C++ framework to achieve high performance. When MS2 spectra replicates are provided, an extra step will be performed to generate consensus spectra across replicates. The consensus spectra are searched against MetaboAnalyst's curated MS2 reference databases for compound identification based on dot product (28) or spectral entropy (30) similarity scores. The complete pipelines for DDA and SWATH-DIA are available from the *Spectra Processing [LC–MS w/wo MS2]* module.

**Figure 2.**
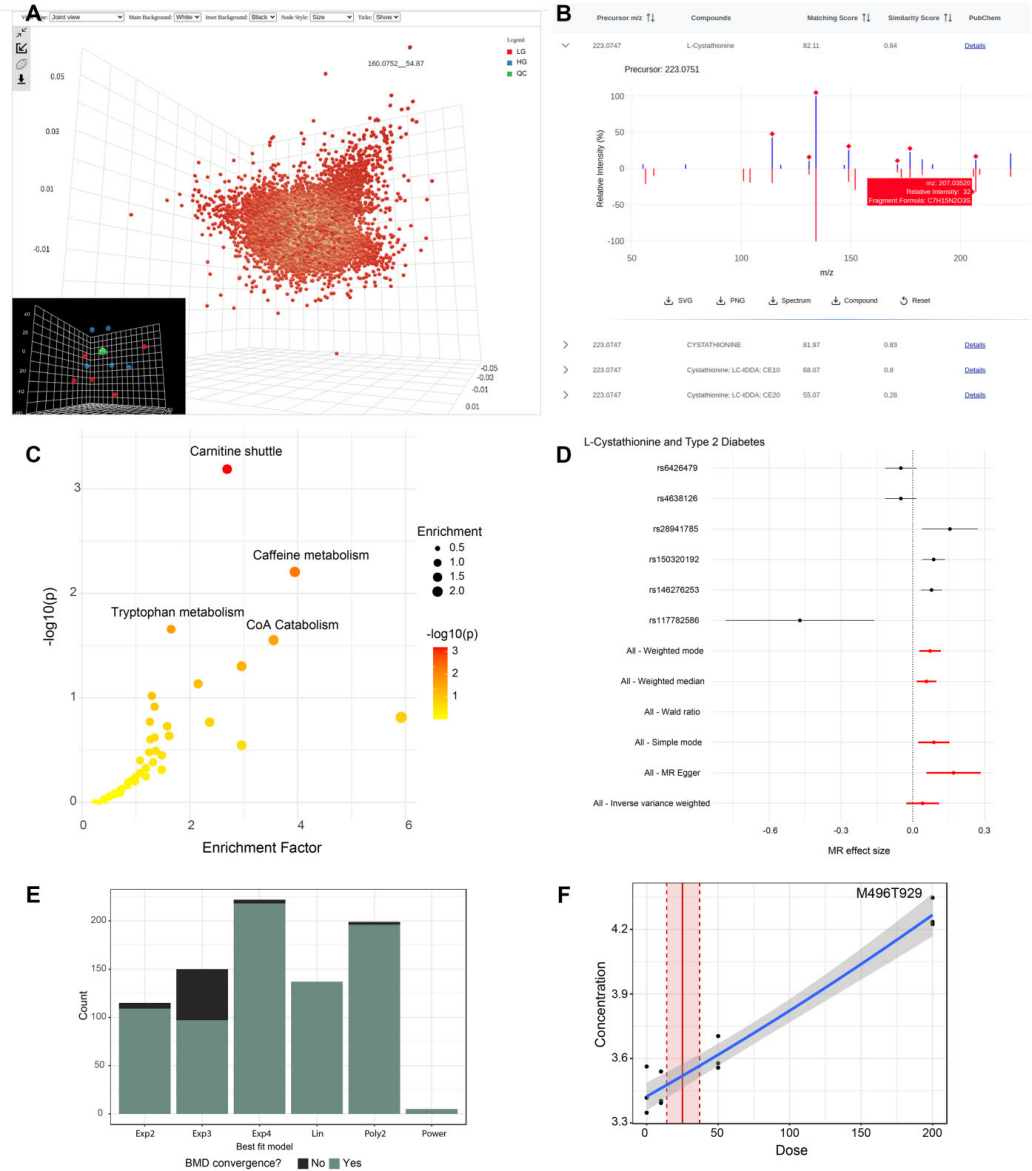
Example outputs from MetaboAnalyst 6.0. (**A**) Integrated 3D PCA score and loading plots summarizing the raw spectra processing results. (**B**) An interactive mirror plot showing the MS2 matching result. Matched fragments are marked with a red diamond. (**C**) Functional analysis results with the top four significant pathways labelled. (**D**) A forest plot comparing the effect sizes calculated based on individual SNPs (black) or using all SNPs by different MR methods (red). (**E**) Bar plots of the dose response curve fitting results showing how many times each model type was identified as the best fit. (**F**) A dose-response curve fitting result showing each of the concentration values (black points), the fitted curve (solid blue line), and the estimated benchmark dose (solid red line) with its lower and upper 95% confidence intervals (dashed red lines), respectively.

Raw spectra must be saved in common open formats and uploaded individually as separate zip files. LC–MS spectra data is mandatory, while MS2 is optional. Upon data uploading, MetaboAnalyst 6.0 first validates the status of the MS files. For SWATH-DIA data, the SWATH window design is automatically extracted from the spectra. If the related information is missing, users will be prompted to manually enter the window design. On the parameters setting page, users can choose the auto-optimized *centWave* algorithm (26) or the *asari* algorithm for LC–MS data processing. If MS2 data is included, spectra deconvolution, consensus, and database searching will be performed using the identified MS features as target list. Once the spectra processing is complete, users can explore both MS and MS2 data processing results (Figure 2A-B) and download the files or directly go to the *Functional Analysis* module.

## MS2 peak annotation

MS2 data could be acquired independently from MS data acquisition. To accommodate this scenario and offer compatibility with MS2 spectra results from other popular tools such as MS-DIAL, we have added a *Peak Annotation [MS2-DDA/DIA]* module to allow users to directly upload MS2 spectra for database searching. Users can enter a single MS2 spectrum or upload an MSP or MGF file containing multiple MS2 spectra. For single spectrum searching, users must specify the *m/z* value of the precursor ion. However, for batch searching based on an MSP file, users do not need to specify the precursors’ *m/z* values. To ensure timely completion of database searching, the public server processes only 20 spectra for each submission (the first 20 spectra by default). Users can manually specify spectra for searching. After conducting this pilot analysis with 20 spectra, users can download the R command history and use our MetaboAnalystR package to annotate all MS2 spectra (26).

Multiple databases are available for compound identification. Database searching can be performed based on regular reference MS2 spectra and/or their corresponding neutral loss spectra. The results are visually summarized as mirror plots based on the matching scores (Figure 2B). Users can interactively explore the MS2 database matching results. The molecular formulas for the MS2 peaks in the reference database spectra are predicted using the BUDDY program (31). Users can download the complete compound identification table together with the mirror plots.

## Causal analysis via two-sample Mendelian randomization

Understanding the causal relationships between metabolites and phenotypes is of great interest in both metabolomics and exposomics. GWAS have established links between genetic variants (e.g. single nucleotide polymorphism, or SNPs) and various phenotypes (32), while recent mGWAS provide connections between genotypes with metabolites or metabolite concentration changes. It becomes possible to estimate causal relationships between metabolites and a phenotype of interest. If a metabolite is causal for a given disease, genetic variants which influence the levels of that metabolite, either directly through affecting related enzymes or indirectly through influencing lifestyle choices (such as dietary habits), should result in a higher risk of the disease. These causal effects can be estimated through Mendelian randomization (MR) analysis (18). MR relies on the principle that genetic variants are randomly distributed across populations, similar to how treatments are randomly assigned in clinical trials. By leveraging this random allocation, MR can evaluate whether a relationship between a metabolite and a phenotype is causal, while reducing the impact of confounding factors and reverse causality that often plague observational studies.

MR analysis in MetaboAnalyst is based on the 2SMR approach (using the *TwoSampleMR* and *MRInstruments* R packages) which enables application of MR methods using summary statistics from non-overlapping individuals (17,33). Users should first select an exposure (i.e. a metabolite) and an outcome (i.e. a disease) of interest. Based on the selections, the program searches for potential instrumental variables (i.e. SNPs) that are associated with both the metabolite from our large collections of the recent mGWAS studies (14) and the disease from the OpenGWAS database (17). The next step is to perform SNP filtering and harmonization to identify independent SNPs through linkage disequilibrium (LD) clumping (34). When SNPs are absent in the GWAS database, proxy SNPs are identified using LD. In addition, it is critical to harmonize SNPs to make sure effect sizes for the SNPs on both exposures and the outcomes are for the same reference alleles. The last step before conducting MR analysis is to exclude SNPs affecting multiple metabolites to reduce horizontal pleiotropy which occurs when a genetic variant influences the outcome through pathways other than the exposure of interest (35). MetaboAnalyst's MR analysis page provides diverse statistical methods (currently 12), each of which has its own strengths and limitations. For instance, the weighted median method is robust to the violation of MR assumptions by some of the genetic variants, while Egger regression method is more robust to horizontal pleiotropy. Users can point their mouse over the corresponding question marks beside each method to learn more details.

## Dose–response analysis

Dose–response analysis is commonly used in toxicology and pharmacology for understanding how varying concentrations of a chemical can impact a biological system. It plays a pivotal role in risk assessment of chemical exposures (36). A key output of dose-response analysis is the benchmark dose (BMD), the minimum dose of a substance that produces a clear, low level health risk relative to the control group (37). Chemicals identified from exposomics are often followed up by dose–response studies to understand their mechanism of action or adverse outcome pathways (21,38,39).

Dose–response experiment design includes a control group (dose = 0) and at least three different dose groups, typically with the same number of replicates in each group. The data should be formatted as a csv file with their dose information included as the second row or column. The analysis workflow consists of four main steps: (i) data upload, integrity checking, processing and normalization; (ii) differential analysis to select features that vary with dose levels; (iii) curve fitting on the intensity or concentration values of those selected features against a suite of linear and non-linear models, and (iv) computing BMD values for each feature. The algorithm for dose–response analysis was adapted from the algorithm we developed for transcriptomics BMD analysis (40,41).

## Updated compound database and knowledge libraries

### Compound database

The compound database has been updated based on HMDB 5.0 (42), with particular efforts made to synchronize with the IDs of other databases such as KEGG (43) and PubChem (44) to improve cross-references during compound mapping and pathway analysis. The compound database was expanded by ∼4000 compounds (after removing ∼10 000 deprecated HMDB entries and adding ∼14 000 new entries).

### MS2 reference spectra database.

A total of 12 MS2 reference databases were collected and curated from public resources, including the HMDB experimental MS2 database (42), the HMDB predicted MS2 database (42), Global Natural Product Social Molecular Networking (GNPS) database (45), MoNA (46), MassBank (46), MINEs (47), LipidBlast (48), RIKEN (49), ReSpect (50), BMDMS (51), VaniyaNP (46) and the MS-DIAL database (v4.90) (52). The complete MS2 reference database currently comprises 10 420 215 MS2 records from 1 551 012 unique compounds. We also created a neutral loss spectra database calculated based on the algorithm implemented by the METLIN neutral loss database (53). The molecular formula of all MS2 fragments were pre-calculated using BUDDY (31).

### Pathway and metabolite set libraries

The KEGG pathway libraries have been updated to their recent version (12/20/2023) via KEGG API. Based on user feedback, the pathway analysis for both targeted and untargeted metabolomics data now supports ∼130 species (up from 28 species in version 5.0), including many new mammals, plants, insects, fungi, and bacteria, etc. We also updated the metabolite set libraries based on HMDB 5.0, MarkerDB (54), as well as manual curation. For instance, a total of 62 metabolite sets associated with dietary and chemical exposures were added during this process. The metabolite set library also incorporated ∼3700 pathways downloaded from the RaMP-DB (55).

## Other features

### Statistical analysis with complex metadata

The *Statistical Analysis [metadata table]* module in MetaboAnalyst 6.0 now provides a comprehensive suite of methods for analyzing and visualizing metabolomics data in relation to various metadata, be it discrete or continuous. Users can quickly assess the correlation patterns among different experimental factors using the metadata overview heatmaps or interactive PCA visualization. The interactive heatmap visualization coupled with hierarchical clustering allows users to easily explore feature abundance variations across different samples and metadata variables. The statistical methods in this module include both univariate linear models with covariate adjustment as well as multivariate methods such as ANOVA Simultaneous Component Analysis (56,57). Random forest is offered for classification with consideration of different metadata variables of interest. More details about this module can be found in our recently published protocol (11).

### Enhanced functional analysis for untargeted metabolomics

Functional analysis of untargeted metabolomics was initially established based on *mummichog* and Gene Set Enrichment Analysis (GSEA) since MetaboAnalyst 4.0 (58). It was further enhanced in MetaboAnalyst 5.0 by incorporating retention time into calculating empirical compounds. MetaboAnalyst 6.0 now allows users to upload an LC–MS peak list along with a corresponding MS2-based compound list to filter out unrealistic empirical compounds to further improve the accuracy in functional analysis (59).

### Enhanced data visualization support

We have enhanced the quality of the interactive and synchronized 3D plots across the dimensionality reduction methods (PCA, PLS-DA, sPLS-DA) used in MetaboAnalyst based on the powerful three.js library (https://threejs.org/). New features include customizable backgrounds, data point annotations and confidence ellipsoids (Figure 2A). We have also implemented interactive plots for clustering heatmaps in the Statistical Analysis modules to better support visual exploration of large data matrices typical in untargeted metabolomics. Both mouse-over and zoom-in functionalities are supported to allow users to examine specific features or patterns of interest. In addition to these enhancements, we also updated the visualization for KEGG’s global metabolic network (43).

## Case study

To illustrate the utility of the new features of MetaboAnalyst 6.0, we used a metabolomics dataset collected in-house that aimed at studying glucose-induced insulin secretion in isolated human islets. The dataset contains five samples of high-glucose (16.7 mM) exposures, five samples of low-glucose (2.8 mM) exposures, both for 30 min, and five quality control (QC) samples. The LC-MS spectra were collected using our Q-Exactive Orbitrap platform (Thermo Scientific, Waltham, MA USA), together with three SWATH-DIA acquisitions from the pooled QC. The spectra were first centroided and converted into mzML format using ProteoWizard (60,61) and uploaded to MetaboAnalyst 6.0. LC–MS spectra processing was performed using the *asari* algorithm. All detected MS1 features were used as a target list for MS2 deconvolution and database searching. A total of 27 209 MS1 features were detected, with 4959 of them identified with at least one potential named chemical identity. Functional analysis using the *mummichog* algorithm indicated compounds showing significant changes between the high-glucose and low-glucose groups were involved in the *Carnitine shuttle*, *Caffeine, Tryptophan*, and *Coenzyme A metabolism* pathways (Figure 2C). These pathways have been consistently identified in previous studies (62–65). Finally, we performed a causal analysis on the associations between one of the significant metabolites identified, *L-Cystathionine* and type 2 diabetes (GWAS ID: finn-b-E4_DM2). The default parameters were used for both SNP filtering and harmonization, as well as MR analysis. Based on these results, a significantly altered cystathionine level was found to have a causal effect on type 2 diabetes (Figure 2D), which aligns well with a study published recently (66). This case study highlights how MetaboAnalyst 6.0 allows users to investigate the chemical identities of MS peaks, elucidate associations between metabolites and phenotypes to unveil previously unknown functional insights. To showcase the dose-response analysis module, we utilized a published data collected from BT549 breast cancer cells treated with four different doses of etomoxir (21). Figure 2E summarizes the results from dose-response modeling. Figure 2F shows an example feature-level BMD calculated based on the fitted curve. The workflow is included as a series of tutorials on our website.

## Comparison with other tools

Several web-based tools have been developed to address various aspects of metabolomics data processing, statistical analysis, functional interpretation, and results visualization. Table 1 compares the main features of MetaboAnalyst 6.0 with other popular tools including the previous version, XCMS online (23), GNPS (45), Workflow4Metabolomics (W4M) (67) and MetExplore (68). For raw data processing, MetaboAnalyst primarily focuses on supporting LC–MS data, whereas W4M also supports GC–MS and NMR raw data processing, and GNPS emphasizes MS2-based compound identification via molecular networks. In comparison, MetaboAnalyst provides an auto-optimized workflow along with an additional algorithm (asari) for efficient LC–MS spectra processing, together with more extensive MS2 spectra libraries for compound identification. In terms of statistical analysis, MetaboAnalyst 6.0 has introduced new modules for dealing with complex metadata, causal analysis and dose–response analysis, while maintaining all other functionalities. MetaboAnalyst contains unique features for enrichment and pathway analysis, and these strengths were further improved in version 6.0, with the addition of unique functions and supports for more species. For network analysis and integration, MetExplore specializes in metabolic network visualization and integration with other omics. These features are addressed by our companion tool, OmicsNet (69). Overall, MetaboAnalyst 6.0 continues to be the most comprehensive tool for metabolomics data processing, analysis and interpretation.

**Table 1.** Comparison of MetaboAnalyst 6.0 with its previous version and other common web-based metabolomics tools. Symbols used for feature evaluations with ‘√’ for present, ‘-’ for absent, and ‘+’ for a more quantitative assessment (more ‘+’ indicate better support)

	MetaboAnalyst					
Tools	6.0	5.0	XCMS Online	GNPS	W4M	MetExplore
**Raw spectra processing**						
MS1 feature detection	+++	++	+	−	+	−
MS1 feature annotation	++	++	++	−	+	−
MS2 spectra deconvolution	√	−	−	−	−	−
MS2 compound identification	+++	−	+++	+++	−	−
Raw spectra results visualization	++	++	+	+	+	−
GC-MS/NMR spectra processing	−	−	−	−	√	−
**Statistical Analysis**						
Univariate	+++	++	+	−	++	−
Multivariate	+++	++	+	−	++	−
Clustering	+++	+++	+	−	+	−
Classification	√	√	−	−	−	−
Complex metadata support	√	−	−	−	−	−
Biomarker analysis	√	√	−	−	−	−
Power analysis	√	√	−	−	−	−
Meta-analysis	√	√	−	−	−	−
Dose-response analysis	√	−	−	−	−	−
Causal analysis	√	−	−	−	−	−
**Functional analysis**						
Functional analysis (MS peaks)	+++	++	++	−	−	−
Enrichment analysis (compounds)	+++	++	−	−	−	+
Functional meta-analysis	√	√	−	−	−	−
Network analysis	++	++	−	+	−	++

• XCMS online: https://xcmsonline.scripps.edu/.

• GNPS: https://gnps.ucsd.edu/.

• Workflow4Metabolomics (W4M): https://workflow4metabolomics.org/.

• MetExplore: https://metexplore.toulouse.inra.fr/metexplore2/

## Conclusion

By incorporating a new MS2 data processing workflow, MetaboAnalyst 6.0 now offers a web-based, end-to-end platform for metabolomics data analysis. The workflow spans from raw MS spectra processing to compound identification to functional analysis. A key motivation in developing version 6.0 was to support the data analysis needs emerging from exposomics and follow-up validation studies. The new statistical analysis module specifically takes into account of complex metadata to better identify robust associations. From these associations, users can perform causal analysis based on 2SMR to narrow down candidate compounds. The remaining compounds can be validated through dose-response studies based on *in vitro* or animal models. Our case study highlights the streamlined analysis workflow from raw spectra processing to compound annotation, to functional interpretation, and finally to causal insights. In conclusion, MetaboAnalyst 6.0 is a user-friendly platform for comprehensive analysis of metabolomics data and help address emerging needs from recent exposomics research. For future directions, we will continue to improve metabolome annotations, better integrate with other omics data, and explore new ways to interact with users via generative artificial intelligence technologies (70–73).

## Data Availability

MetaboAnalyst 6.0 is freely available at https://www.metaboanalyst.ca. No log in required.
